# Prognostic value of the cardiometabolic index for cardiovascular outcomes in patients with type 2 diabetes mellitus

**DOI:** 10.3389/fnut.2026.1787383

**Published:** 2026-07-24

**Authors:** Cheng Zeng, Maojun Liu, Peiqi Tang, Ying Xin, Xinqun Hu

**Affiliations:** 1 Department of Cardiology, The Second Xiangya Hospital of Central South University, Changsha, Hunan, China; 2 Department of Geriatrics, The Second Xiangya Hospital of Central South University, Changsha, Hunan, China

**Keywords:** all-cause mortality, cardiometabolic index, cardiovascular outcome, major adverse cardiovascular events, type 2 diabetes mellitus

## Abstract

**Background:**

The Cardiometabolic Index (CMI) serves as a measurable indicator capable of concurrently reflecting the presence of metabolic disorders and the status of central obesity. The aim of this study is to evaluate the association between the CMI and the risk of developing major adverse cardiovascular events (MACEs) and all-cause mortality in patients with type 2 diabetes mellitus (T2DM).

**Methods:**

This *post-hoc* analysis included 10,094 participants from the Action to Control Cardiovascular Risk in Diabetes (ACCORD) study and the ACCORD Follow-up study with baseline CMI data. The natural logarithm (ln) of each CMI measurement was computed. Univariate and multivariate Cox proportional hazard regression analyses were conducted to examine the relationship between CMI and the risk of MACEs and all-cause mortality. The incremental predictive value of CMI was further assessed.

**Results:**

Over a median follow-up period of 8.82 years, 1,791 (17.74%) participants experienced MACEs, and 1,914 (18.96%) all-cause mortality were recorded. After adjusting for traditional cardiovascular risk factors, multivariate Cox proportional hazards regression analysis demonstrated a significant association between CMI and the risk of MACEs and all-cause mortality. Incorporating CMI into the conventional risk model improved the predictive efficacy for MACEs and all-cause mortality.

**Conclusions:**

Elevated CMI is associated with an increased risk of MACEs and all-cause mortality in patients with T2DM. Furthermore, CMI improves the predictive performance for MACEs and all-cause mortality risk in this patient population.

## Introduction

Diabetes mellitus is one of the major chronic non-communicable diseases threatening human health globally. Its pathogenesis is complex, involving the combined effects of multiple factors, including genetic predisposition and environmental influences (1). Approximately 90% to 95% of all diabetes cases are classified as Type 2 Diabetes Mellitus (T2DM) (2, 3). A 2019 comprehensive analysis estimated that diabetes mellitus ranks as the eighth leading cause of death and disability worldwide (4). Furthermore, in 2021, the global prevalence of confirmed diabetes cases reached 537 million, with associated disease management expenditures totaling USD 966 billion (5). The International Diabetes Federation (IDF) projects that by 2045, the global diabetic population will rise to 783 million, with related medical expenditures anticipated to exceed USD 1.054 trillion (6). Cardiovascular disease (CVD)—encompassing peripheral artery disease (PAD), cerebrovascular disease, coronary artery disease (CAD), and heart failure—represents the most prevalent complication in patients with T2DM and the primary driver of morbidity and mortality in this population (7, 8). Contemporary understanding of T2DM as a heterogeneous disease has advanced progressively. CVD risk in this context is influenced by multiple interacting factors, underscoring the urgent need to prioritize individualized management strategies and conduct timely, cutting-edge evaluations in this domain.

Patients with T2DM typically present with insulin resistance (IR), obesity, and dyslipidemia—all well-established, independent risk factors for the development of various CVDs (9–13). The triglyceride-to-high-density lipoprotein cholesterol ratio (TG/HDL-C) functions as a robust surrogate marker for IR and a reliable predictor of CVD risk (14). The waist-to-height ratio (WHtR), calculated from waist circumference (WC) and height, more accurately identifies abdominal obesity and assesses cardiometabolic risk compared to body mass index (BMI) (15, 16). In 2015, Wakabayashi et al. proposed a novel composite indicator, the cardiometabolic index (CMI), which integrates parameters for assessing lipid metabolism and abdominal obesity. It is calculated as the product of the WHtR and the TG/HDL-C (17). Compared with single metrics including TG/HDL-C and WHtR, CMI achieves superior predictive performance for coronary heart disease. Its multiplicative construction captures synergistic risks undetectable by separate components, highlighting the incremental prognostic value of this composite marker (18). The CMI, a metabolism-related marker, was initially developed to predict diabetes mellitus risk. Emerging evidence indicates that CMI is additionally associated with the risk of hypertension, heart failure, non-alcoholic fatty liver disease, major depressive disorder, chronic obstructive pulmonary disease, as well as with all-cause and cardiovascular mortality (17, 19–25). However, to date, no studies have investigated the association between the CMI and major adverse cardiovascular events (MACEs) in patients with T2DM.

This study aims to investigate the association between the CMI and the risk of MACEs as well as all-cause mortality in patients with T2DM, using data from the Action to Control Cardiovascular Risk in Diabetes (ACCORD) clinical trial and the ACCORD follow-up study (ACCORDION).

## Materials and methods

### Study design and participants

This study conducted a *post-hoc* analysis using data from the ACCORD/ACCORDION trial, which is registered on ClinicalTrials.gov under identifier NCT00000620. The study's underlying rationale, experimental design, and primary outcome measures have been detailed in prior publications (26, 27). Briefly, a total of 10,251 participants with T2DM aged 40–79 were enrolled in the ACCORD/ACCORDION trial. Participants had a median glycated hemoglobin (HbA1c) level of 8.1% and a mean age of 62.2 years. Every individual in the trial either had a history of cardiovascular disease or was at heightened risk of developing it. Between June 2001 and June 2009, 77 sites across North America served as study locations. As noted in the ACCORD research findings, 96% of participants had completed critical status follow-up by June 30, 2009, with partial data available for the remaining individuals. Participants received treatment and were followed for an average duration of 5 years. From 2011 to 2014, ACCORD patients who joined the ACCORDION extension completed an extra 3.5 years of follow-up on average, via outpatient consultations and telephone check-ins.

### Data collection and outcomes

**Figure 1:**
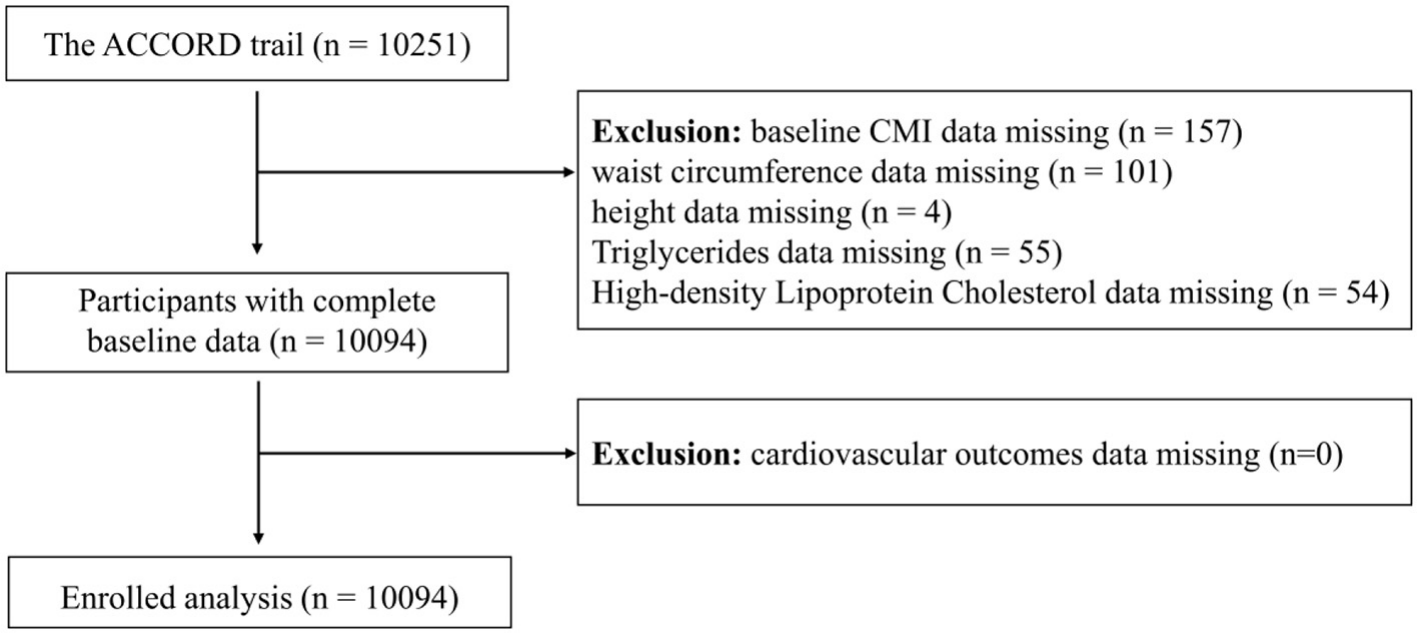
Flowchart of this study.

The analyzed dataset encompassed demographic traits and clinical attributes: age, sex, racial background, educational level, medical history, physical exam results, prior medication regimens, and laboratory measurements. These measurements included HbA1c, total cholesterol (TC), triglycerides (TG), high-density lipoprotein cholesterol (HDL-C), low-density lipoprotein cholesterol (LDL-C), very low-density lipoprotein (VLDL), alanine aminotransferase (ALT), estimated glomerular filtration rate (eGFR), serum creatinine, urinary albumin, and urinary creatinine. The CMI was calculated using the formula: CMI = TG (mmol/L) / HDL-C (mmol/L) × WC (cm) / height (cm). Of the 10,251 participants, 157 lacked baseline data on CMI; thus, 10,094 participants were ultimately included in the analysis (Figure 1).

The main endpoint of the present study was the incidence of MACEs. These events were defined as a composite outcome including CVD mortality, nonfatal myocardial infarction (MI), and nonfatal stroke. All-cause mortality was specified as the secondary endpoint.

### Statistical analysis

Statistical analyses were carried out with three software tools: SPSS 26.0 (IBM Corp., Armonk, NY, USA), R (The R Foundation for Statistical Computing, Vienna, Austria), and EmpowerStats (X&Y Solutions, Inc., Boston, MA, USA). Since the CMI exhibited a skewed distribution, all corresponding measurements underwent natural logarithm (ln) transformation. This process enhances the symmetry of the distribution and reduces the impact of outlier values. Because CMI values can be less than 1—leading to negative ln results—measurements were first multiplied by 10 before the transformation was applied. This linear rescaling exerts no influence on the interpretation of derived hazard ratios and facilitates clinical translation of the analytical findings.

Baseline traits are reported as mean ± standard deviation (SD), frequency (*n*, %), or median (interquartile range [IQR])—with the choice determined by each variable's distribution. One-way analysis of variance (ANOVA) or the Kruskal-Wallis test was used to compare continuous variables. For categorical variables, comparisons were conducted using the chi-square test.

Kaplan-Meier estimation was used to assess cumulative event rates for MACEs and all-cause mortality, with stratification based on ln (10 × CMI) quantiles. Log-rank tests were applied to compare discrepancies in these hazard estimates. Hazard ratios (HRs) and their respective 95% confidence intervals (CIs) for MACEs were computed using Cox proportional hazards regression models. Before performing multivariate Cox regression, univariable analysis was conducted to screen for potential confounders and identify variables significantly associated with MACEs. Covariate selection was guided by both statistical criteria and clinical relevance. Variables demonstrating statistical significance in univariable analysis (*p* < 0.1; Table S1) were entered into the multivariable model. Additionally, clinically meaningful variables strongly linked to MACEs and those exhibiting an estimated effect modification exceeding 10% were incorporated. To mitigate residual confounding, each analysis employed an unadjusted model alongside three hierarchically adjusted models with incremental covariate adjustment. Model 1 was adjusted for potential confounders including age, sex, race, education, living alone, depression, CVD history, family history of heart disease, heart attack, or stroke, heart failure, previous hypertension, duration of diabetes, as well as smoking status. On the basis of Model 1, Model 2 was further adjusted for systolic blood pressure (SBP), diastolic blood pressure (DBP), HbA1c, TC, ALT, serum creatinine, eGFR, and urinary albumin levels. After incorporating all covariates in Model 2, Model 3 additionally accounted for the use of diuretics, calcium channel blockers (CCB), beta-blockers, sulfonylureas, biguanides, thiazolidinediones, insulin preparations, and statins. Multicollinearity diagnostics were performed across all covariates incorporated into the regression analyses. As commonly accepted cutoff criteria, multicollinearity is considered evident when the tolerance value falls below 0.1 or the variance inflation factor (VIF) exceeds 5. Collinearity assessment outcomes revealed that all included variables yielded VIF values < 5 alongside tolerance values above 0.1, which verified the absence of obvious multicollinearity among these confounding factors (Table S2). The Schoenfeld residual-based test was adopted to verify the proportional hazards (PH) assumption of the Cox regression model. Global and variable-specific test results suggested no statistically significant deviation from the PH assumption (*P* > 0.05), implying that hazard ratios of included covariates remained constant across follow-up time and the PH assumption was satisfied for all analyzed covariates.

Moreover, the incremental predictive utility of the CMI beyond standard risk factors was evaluated using C-statistics, net reclassification improvement (NRI), and integrated discrimination improvement (IDI). Subsequent calibration analyses were performed to evaluate whether the incorporation of CMI improved the clinical predictive utility of the baseline risk model.

Subgroup and interaction analyses were performed, with stratification based on sex, age (< 65 years and ≥65 years), race, CVD history, previous hyperlipidemia, previous hypertension, smoking, duration of diabetes (< 10 years and ≥10 years), BMI (< 25 kg/m^2^ and ≥25 kg/m^2^), HbA1c (< 8.1% and ≥8.1%), eGFR (< 60 mL/min/1.73 m^2^, ≥60 mL/min/1.73 m^2^, < 90 mL/min/1.73 m^2^, and ≥90 mL/min/1.73 m^2^), and insulin use.

Sensitivity analyses were conducted to verify the robustness of the present findings. Receiver operating characteristic (ROC) curve analysis was performed to determine the optimal cut-off value of CMI for clinical application.

## Results

### Baseline characteristics stratified by tertiles of ln (10 × CMI)

A total of 10,094 participants included in this analysis were divided into three tertiles according to ln (10 × CMI) values. Table 1 outlines the baseline characteristics of the study cohort. Around 61.48% of the individuals were male, with an average age of 62.80 ± 6.64 years, and 62.43% self-identified as White. The ranges for ln (10 × CMI) across tertiles were as follows: low (0.28–2.87), middle (2.87–3.51), and high (3.51–7.30). These correspond to CMI values of 0.13–1.76, 1.76–3.33, and 3.33–148.76, in that order. Participants in the higher tertiles of ln (10 × CMI) had a greater likelihood of being male and White. They were also more prone to having a history of CVD or heart failure, reporting a family history of heart disease, heart attack, or stroke, and having pre-existing hyperlipidemia, shorter diabetes duration, or proteinuria. Additionally, these individuals tended to show higher BMI, DBP, heart rate, HbA1c, TG, VLDL, ALT, and serum creatinine levels. They also had lower HDL-C and eGFR levels, and were more likely to use diuretics, CCBs, beta-blockers, sulfonylureas, biguanides, insulin, and statins.

**Table 1:** Baseline characteristics of participants by tertiles of ln (10 × CMI).

Characteristics	Low (*n* = 3,365)	Middle (*n* = 3,364)	High (*n* = 3,365)	*P*-value
CMI	1.17 (0.85–1.44)	2.44 (2.09–2.84)	5.02 (4.04–6.95)	< 0.001
Ln (10 × CMI)	2.36 (0.40)	3.19 (0.18)	4.03 (0.46)	< 0.001
Age (years)	63.47 (6.83)	63.01 (6.68)	61.92 (6.31)	< 0.001
Sex, *n* (%)				< 0.001
Male	1,879 (55.84%)	2,085 (61.98%)	2,242 (66.63%)	
Female	1,486 (44.16%)	1,279 (38.02%)	1,123 (33.37%)	
Race, *n* (%)				< 0.001
White	1,556 (46.24%)	2,183 (64.89%)	2,563 (76.17%)	
Non-white	1,809 (53.76%)	1,181 (35.11%)	802 (23.83%)	
Treatment, *n* (%)				0.349
Standard glucose control	1,683 (50.01%)	1,711 (50.86%)	1,652 (49.09%)	
Intensive glucose control	1,682 (49.99%)	1,653 (49.14%)	1,713 (50.91%)	
Education, *n* (%)				< 0.001
Less than high school graduate	573 (17.04%)	511 (15.19%)	408 (12.14%)	
High school grad (or GED)	910 (27.07%)	844 (25.10%)	908 (27.01%)	
Some college or technical school	993 (29.54%)	1,144 (34.02%)	1,174 (34.92%)	
College graduate or more	886 (26.35%)	864 (25.69%)	872 (25.94%)	
Living alone, *n* (%)	2,630 (78.18%)	2,704 (80.40%)	2,719 (80.80%)	0.016
Depression, *n* (%)	625 (18.58%)	800 (23.79%)	969 (28.80%)	< 0.001
Smoking, *n* (%)	449 (13.34%)	417 (12.40%)	541 (16.08%)	< 0.001
CVD history, *n* (%)	1,059 (31.47%)	1,186 (35.26%)	1,311 (38.96%)	< 0.001
Family history of heart disease, heart attack, or stroke, *n* (%)	1,515 (45.04%)	1,610 (47.86%)	1,718 (51.05%)	< 0.001
Heart failure, *n* (%)	123 (3.66%)	155 (4.61%)	206 (6.12%)	< 0.001
Previous hyperlipidemia, *n* (%)	2,233 (66.36%)	2,371 (70.48%)	2,461 (73.14%)	< 0.001
Previous hypertension, *n* (%)	2,549 (75.75%)	2,494 (74.14%)	2,566 (76.26%)	0.109
Duration of diabetes (years)	10.00 (6.00–17.00)	9.00 (5.00–15.00)	8.00 (5.00–13.00)	< 0.001
Proteinuria, *n* (%)	598 (17.78%)	662 (19.68%)	741 (22.02%)	< 0.001
Weight (kg)	87.34 (17.54)	94.44 (17.85)	98.68 (17.97)	< 0.001
Height (cm)	169.20 (9.89)	170.23 (9.78)	171.09 (9.58)	< 0.001
WC (cm)	101.18 (13.08)	107.81 (13.02)	111.22 (12.85)	< 0.001
BMI (kg/m^2^)	30.47 (5.40)	32.52 (5.22)	33.63 (5.09)	< 0.001
SBP (mmHg)	137.37 (17.22)	135.85 (16.75)	135.86 (17.30)	< 0.001
DBP (mmHg)	74.36 (10.58)	74.71 (10.57)	75.59 (10.80)	< 0.001
Heart rate, bpm	72.25 (11.59)	72.19 (11.64)	73.54 (11.94)	< 0.001
HbA1C (%)	8.27 (1.05)	8.27 (1.05)	8.36 (1.07)	< 0.001
TC (mg/dL)	174.49 (36.84)	179.24 (38.17)	196.14 (46.72)	< 0.001
TG (mg/dL)	91.00 (72.00–110.00)	154.00 (132.00–182.00)	271.00 (220.00–349.00)	< 0.001
VLDL (mg/dL)	18.00 (14.00–22.00)	31.00 (26.00–36.00)	54.00 (44.00–66.00)	< 0.001
LDL-C (mg/dL)	104.83 (31.26)	106.77 (33.58)	102.97 (36.53)	< 0.001
HDL-C (mg/dL)	51.07 (12.33)	40.69 (7.50)	33.83 (6.86)	< 0.001
ALT (mg/dL)	22.00 (17.00–29.00)	24.00 (18.00–33.00)	26.00 (20.00–35.00)	< 0.001
Serum creatinine (mg/dL)	0.90 (0.22)	0.91 (0.23)	0.92 (0.25)	0.266
eGFR (ml/min/1.73 m^2^)	90.40 (77.10–106.10)	89.20 (74.20–104.10)	89.30 (73.30–105.20)	< 0.001
Urinary albumin (mg/dL)	1.36 (0.63–3.99)	1.54 (0.73–4.63)	1.91 (0.83–6.78)	< 0.001
Urinary creatinine (g/dL)	112.05 (77.30–159.00)	116.80 (81.70–161.50)	114.70 (79.70–154.85)	0.028
Medications, *n* (%)				
Diuretics	1,169 (34.74%)	1,216 (36.15%)	1,307 (38.84%)	0.002
ARB/ACEI	2,321 (68.97%)	2,333 (69.35%)	2,338 (69.48%)	0.897
CCB	693 (20.59%)	615 (18.28%)	621 (18.45%)	0.027
Beta-blockers	804 (23.92%)	1,059 (31.57%)	1,178 (35.14%)	< 0.001
Sulfonylureas	1,657 (49.24%)	1,860 (55.29%)	1,884 (56.00%)	< 0.001
Biguanides	2,031 (60.36%)	2,237 (66.50%)	2,187 (65.01%)	< 0.001
Meglitinides	84 (2.50%)	84 (2.50%)	83 (2.47%)	0.996
Thiazolidinediones	779 (23.15%)	734 (21.82%)	715 (21.25%)	0.157
Insulins	1,349 (40.09%)	1,129 (33.56%)	1,044 (31.03%)	< 0.001
Statins	2,212 (65.99%)	2,196 (65.53%)	2,009 (59.90%)	< 0.001
Fibrates	80 (2.39%)	175 (5.23%)	359 (10.72%)	< 0.001
Cholesterol absorption inhibitors	68 (2.03%)	66 (1.97%)	71 (2.12%)	0.911
MACEs, *n* (%)	491 (14.59%)	606 (18.01%)	694 (20.62%)	< 0.001
CVD mortality	162 (4.81%)	220 (6.54%)	274 (8.14%)	< 0.001
Nonfatal MI	248 (7.37%)	309 (9.19%)	367 (10.91%)	< 0.001
Nonfatal stroke	135 (4.01%)	162 (4.82%)	179 (5.32%)	0.038
All-cause mortality, *n* (%)	556 (16.52%)	622 (18.49%)	736 (21.87%)	< 0.001

Data are showed as mean (standard deviations), median (Q1–Q3), or as n (%). The tertile ranges of ln (10 × CMI) were low (0.28–2.87), middle (2.87–3.51), and high (3.51–7.30). *P* value for the test of the difference across tertiles of ln (10 × CMI) were obtained by using the χ2 test (categorical variables), ANOVA (continuous variables), or Kruskal-Wallis test (nonparametric comparisons). ALT, alanine aminotransferase; ARB, angiotensin receptor blocker; ACEI, angiotensin converting enzyme inhibitors; BMI, body mass index; CCB, calcium channel blockers; CMI, cardiometabolic index; CVD, cardiovascular disease; DBP, diastolic blood pressure; eGFR, estimated glomerular filtration rate; GED, general education development; HDL-C, high-density lipoprotein cholesterol; LDL-C, low-density lipoprotein cholesterol; MACEs, major adverse cardiovascular events; MI, myocardial infarction; SBP, systolic blood pressure; TC, total cholesterol; TG, triglycerides; VLDL, very low-density lipoprotein; WC, waist circumference.

### The relationship between ln (10 × CMI) and outcomes

Participants in the high tertile of ln (10 × CMI) faced a heightened risk of MACEs and all-cause mortality (Table 1). Across the entire study cohort, 1,791 individuals (17.74%) experienced MACEs during a median follow-up period of 8.82 years. These events included 656 cases of CVD mortality (6.5%), 924 nonfatal MIs (9.15%), and 476 nonfatal strokes (4.72%). In total, 1,914 deaths were recorded, accounting for 18.96% of all participants.

**Figure 2:**
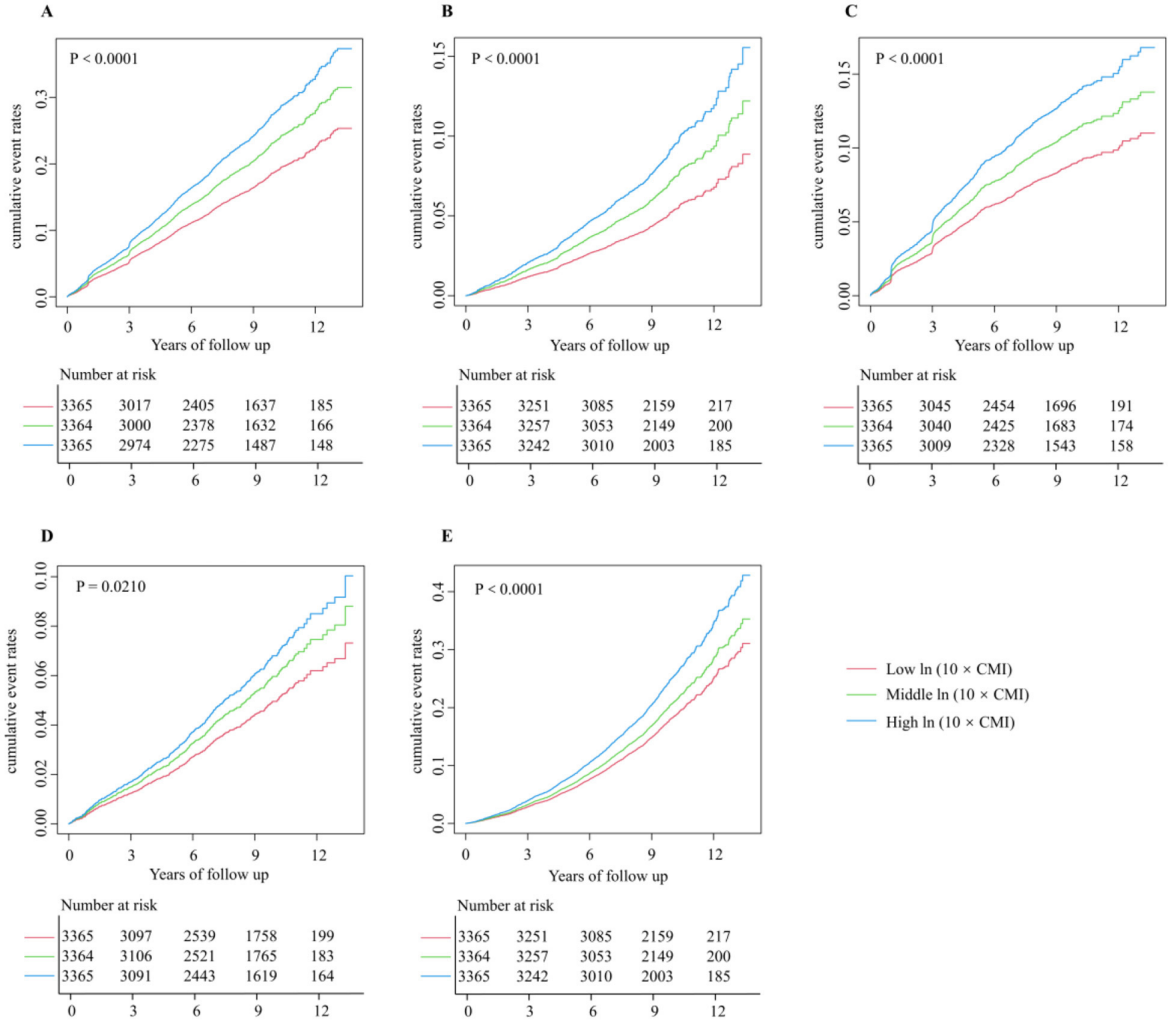
Kaplan–Meier curves for MACEs and all-cause mortality. Middle and high ln (10 × CMI) vs. low ln (10 × CMI). **(A)** MACEs; **(B)** CVD mortality; **(C)** Nonfatal MI; **(D)** Nonfatal stroke; **(E)** All-cause mortality. CMI, cardiometabolic index; CVD, cardiovascular disease; MACEs, major adverse cardiovascular events; MI, myocardial infarction.

Cumulative event rates of MACEs—a composite of CVD mortality, nonfatal MI, and nonfatal stroke—and all-cause mortality were evaluated via Kaplan-Meier curves (Figure 2), which the log-rank test revealed to have statistically significant differences (*P* < 0.05) when stratified by ln (10 × CMI) tertiles. Specifically, participants in the higher tertiles of ln (10 × CMI) demonstrated a greater cumulative event rates than their counterparts in the lower tertiles, a pattern that underscored the incremental association between elevated ln (10 × CMI) values and increased long-term cardiovascular and all-cause mortality risk.

Three multivariate Cox proportional hazards regression models were employed to investigate the relationship between ln (10 × CMI) and outcome events (Table 2). Model 1 included adjustments for age, sex, race, education, living alone, depression, CVD history, family history of heart disease, heart attack, or stroke, heart failure, previous hypertension, duration of diabetes, as well as smoking status. Building on Model 1, Model 2 was further adjusted to account for SBP, DBP, HbA1c, TC, ALT, serum creatinine, eGFR, and urinary albumin levels. Model 3 expanded upon Model 2 by adding medication use—encompassing diuretics, CCBs, beta-blockers, sulfonylureas, biguanides, thiazolidinediones, insulin, and statins—as an extra covariate. Even after thorough adjustment for potential confounding variables in Model 3, higher ln (10 × CMI) showed a significant association with elevated cumulative risk of MACEs (highest tertile: HR 1.26, 95% CI: 1.11–1.43, *P* = 0.0005). Specifically, each 1 SD rise in ln (10 × CMI) corresponded to a 9% increase in MACEs risk (HR 1.09, 95% CI: 1.03–1.15, *P* = 0.0017). Furthermore, baseline ln (10 × CMI) retained a significant association with CVD mortality, while no meaningful link was detected between baseline ln (10 × CMI) and nonfatal stroke. Additionally, baseline ln (10 × CMI) was significantly correlated with all-cause mortality (highest tertile: HR 1.29, 95% CI: 1.14–1.46, *P* < 0.0001), with each 1 SD increase in ln (10 × CMI) leading to a 10% higher risk of all-cause mortality (HR 1.10, 95% CI: 1.04–1.16, *P* = 0.0003). Collectively, these results indicate that ln (10 × CMI) may function as a predictive marker for MACEs and all-cause mortality in patients with T2DM.

**Table 2:** Risk of MACEs and all-cause mortality based on ln (10 × CMI).

Outcome	Events/n	Non-adjusted		Model 1		Model 2		Model 3	
		HR (95% CI)	*P*-value	HR (95% CI)	*P*-value	HR (95% CI)	*P*-value	HR (95% CI)	*P*-value
MACEs									
Ln (10 × CMI)		1.23 (1.16, 1.30)	< 0.0001	1.20 (1.12, 1.28)	< 0.0001	1.14 (1.06, 1.22)	0.0002	1.12 (1.04, 1.20)	< 0.0001
Low	491/3365	Ref		Ref		Ref		Ref	
Middle	606/3364	1.24 (1.10, 1.40)	0.0003	1.19 (1.05, 1.35)	0.0048	1.16 (1.03, 1.31)	0.0180	1.15 (1.01, 1.30)	0.0304
High	694/3365	1.47 (1.31, 1.65)	< 0.0001	1.40 (1.24, 1.58)	< 0.0001	1.29 (1.14, 1.47)	< 0.0001	1.26 (1.11, 1.43)	0.0005
Per 1 SD		1.17 (1.12, 1.23)	< 0.0001	1.15 (1.10, 1.21)	< 0.0001	1.10 (1.05, 1.16)	0.0002	1.09 (1.03, 1.15)	0.0017
*P* for trend			< 0.0001		< 0.0001		< 0.0001		0.0006
CVD mortality									
Ln (10 × CMI)		1.28 (1.17, 1.41)	< 0.0001	1.27 (1.15, 1.42)	< 0.0001	1.22 (1.09, 1.37)	0.0005	1.19 (1.06, 1.34)	< 0.0001
Low	162/3365	Ref		Ref		Ref		Ref	
Middle	220/3364	1.38 (1.12, 1.68)	0.0021	1.35 (1.10, 1.67)	0.0043	1.29 (1.05, 1.59)	0.0171	1.25 (1.01, 1.55)	0.0385
High	274/3365	1.75 (1.44, 2.13)	< 0.0001	1.73 (1.40, 2.12)	< 0.0001	1.61 (1.30, 1.99)	< 0.0001	1.54 (1.24, 1.91)	0.0001
Per 1 SD		1.21 (1.13, 1.31)	< 0.0001	1.21 (1.11, 1.31)	< 0.0001	1.17 (1.07, 1.28)	0.0005	1.15 (1.05, 1.26)	0.0026
*P* for trend			< 0.0001		< 0.0001		< 0.0001		< 0.0001
Nonfatal MI									
ln (10 × CMI)		1.28 (1.18, 1.39)	< 0.0001	1.23 (1.12, 1.34)	< 0.0001	1.17 (1.06, 1.28)	0.0014	1.16 (1.05, 1.27)	0.0032
Low	248/3365	Ref		Ref		Ref		Ref	
Middle	309/3364	1.25 (1.06, 1.48)	0.0084	1.16 (0.98, 1.38)	0.0829	1.15 (0.97, 1.37)	0.1149	1.15 (0.96, 1.37)	0.1210
High	367/3365	1.53 (1.30, 1.79)	< 0.0001	1.39 (1.17, 1.64)	0.0002	1.30 (1.09, 1.56)	0.0034	1.29 (1.08, 1.55)	0.0055
Per 1 SD		1.21 (1.14, 1.29)	< 0.0001	1.17 (1.09, 1.26)	< 0.0001	1.13 (1.05, 1.21)	0.0014	1.12 (1.04, 1.20)	0.0032
*P* for trend			< 0.0001		0.0002		0.0033		0.0054
Nonfatal stroke									
ln (10 × CMI)		1.16 (1.03, 1.30)	0.0127	1.15 (1.01, 1.30)	0.0287	1.09 (0.95, 1.24)	0.2114	1.07 (0.94, 1.23)	0.2927
Low	135/3365	Ref		Ref		Ref		Ref	
Middle	162/3364	1.20 (0.96, 1.51)	0.1124	1.19 (0.94, 1.50)	0.1465	1.17 (0.92, 1.48)	0.1925	1.18 (0.93, 1.50)	0.1693
High	179/3365	1.37 (1.10, 1.71)	0.0056	1.35 (1.06, 1.71)	0.0135	1.24 (0.97, 1.59)	0.0831	1.22 (0.95, 1.57)	0.1163
Per 1 SD		1.12 (1.02, 1.22)	0.0127	1.11 (1.01, 1.22)	0.0287	1.07 (0.96, 1.18)	0.2114	1.06 (0.95, 1.17)	0.2927
*P* for trend			0.0056		0.0136		0.0862		0.1231
All-cause mortality									
ln (10 × CMI)		1.17 (1.11, 1.24)	< 0.0001	1.18 (1.11, 1.26)	< 0.0001	1.14 (1.07, 1.22)	< 0.0001	1.13 (1.06, 1.21)	0.0003
Low	556/3365	Ref		Ref		Ref		Ref	
Middle	622/3364	1.14 (1.01, 1.27)	0.0295	1.14 (1.01, 1.28)	0.0304	1.11 (0.99, 1.25)	0.0760	1.11 (0.98, 1.25)	0.0898
High	736/3365	1.38 (1.23, 1.54)	< 0.0001	1.39 (1.23, 1.56)	< 0.0001	1.32 (1.16, 1.49)	< 0.0001	1.29 (1.14, 1.46)	< 0.0001
Per 1 SD		1.13 (1.08, 1.18)	< 0.0001	1.14 (1.09, 1.19)	< 0.0001	1.11 (1.05, 1.17)	< 0.0001	1.10 (1.04, 1.16)	0.0003
*P* for trend			< 0.0001		< 0.0001		< 0.0001		< 0.0001

Model 1: adjusted for age, sex, race, education, living alone, depression, CVD history, family history of heart disease, heart attack, or stroke, heart failure, previous hypertension, duration of diabetes, smoking.

Model 2: adjusted for model 1 covariables plus SBP, DBP, HbA1C, TC, ALT, serum creatinine, eGFR, urinary albumin.

Model 3: adjusted for model 2 covariables plus the medications use, diuretics, CCB, beta-blockers, sulfonylurea, biguanides, thiazolidinediones, insulins, and statin.

### Subgroup analyses

**Figure 3:**
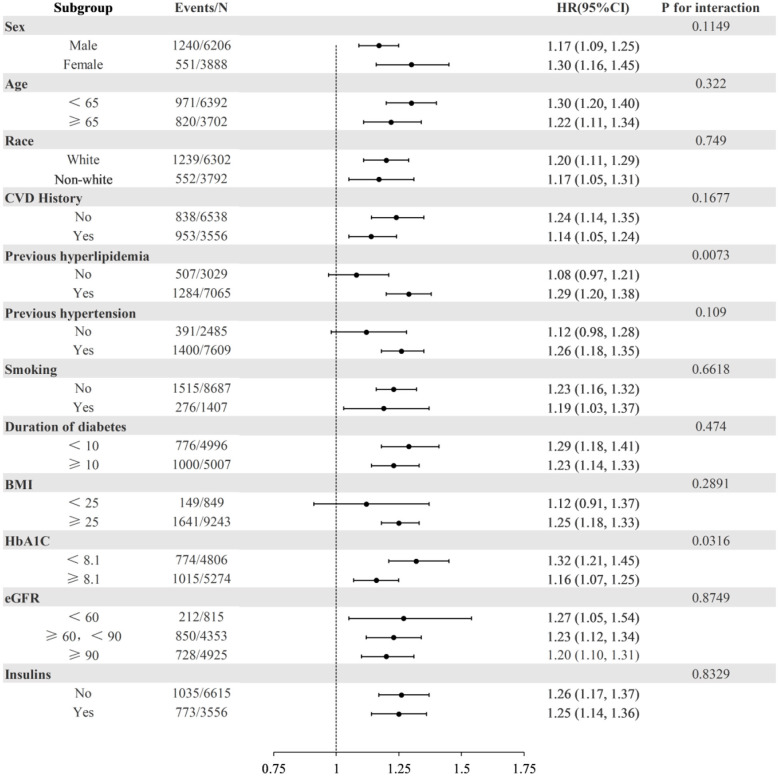
Subgroup and interaction analyses of the association between ln (10 × CMI) and the risk of MACEs. Participants were stratified by sex, age (< 65 years and ≥ 65 years), race, CVD history, previous hyperlipidemia, previous hypertension, smoking status, duration of diabetes (< 10 years and ≥ 10 years), BMI (< 25 kg/m^2^ and ≥ 25 kg/m^2^), HbA1C (< 8.1% and ≥ 8.1%), eGFR (< 60 ml/min/1.73 m^2^, ≥ 60 ml/min/1.73 m^2^, < 90 ml/min/1.73 m^2^, and ≥ 90 ml/min/1.73 m^2^), and insulin use. BMI, body mass index; CMI, cardiometabolic index; CVD, cardiovascular disease; eGFR, estimated glomerular filtration rate; MACEs, major adverse cardiovascular events.

**Figure 4:**
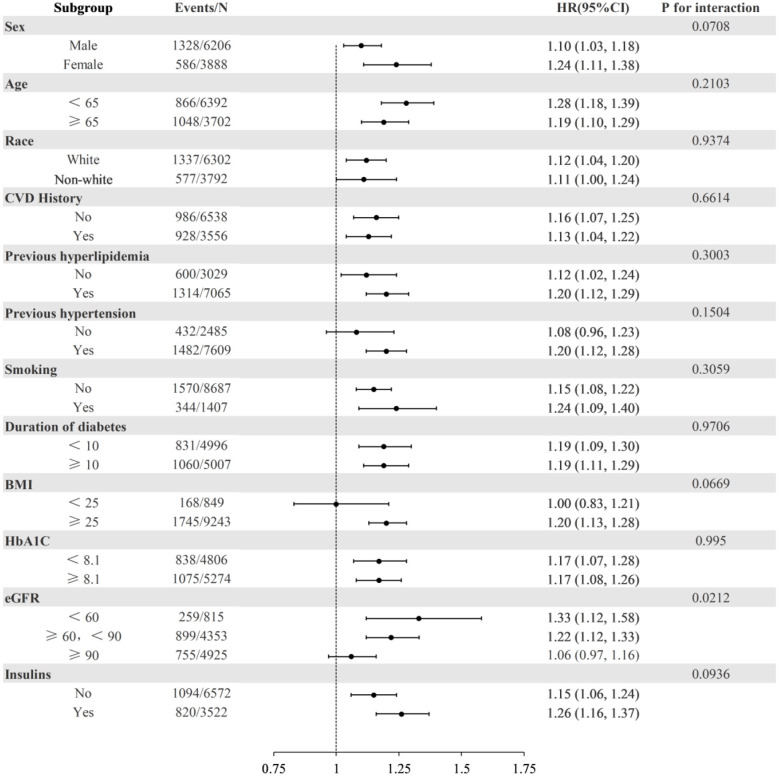
Subgroup and interaction analyses of the association between ln (10 × CMI) and the risk of all-cause mortality. Participants were stratified by sex, age (< 65 years and ≥ 65 years), race, CVD history, previous hyperlipidemia, previous hypertension, smoking status, duration of diabetes (< 10 years and ≥ 10 years), BMI (< 25 kg/m^2^ and ≥ 25 kg/m^2^), HbA1C (< 8.1% and ≥ 8.1%), eGFR (< 60 ml/min/1.73 m^2^, ≥ 60 ml/min/1.73 m^2^, < 90 ml/min/1.73 m^2^, and ≥ 90 ml/min/1.73 m^2^), and insulin use. BMI, body mass index; CMI, cardiometabolic index; CVD, cardiovascular disease; eGFR, estimated glomerular filtration rate.

To delve deeper into the relationship between ln (10 × CMI) and outcome events, subgroup analyses were performed. These analyses were stratified by sex, age (split into < 65 years and ≥ 65 years), race, CVD history, previous hyperlipidemia, previous hypertension, smoking status, diabetes duration (categorized as < 10 years and ≥ 10 years), BMI (< 25 kg/m^2^ and ≥ 25 kg/m^2^), HbA1C (< 8.1% and ≥ 8.1%), eGFR (< 60 ml/min/1.73 m^2^, ≥ 60 ml/min/1.73 m^2^, < 90 ml/min/1.73 m^2^, and ≥ 90 ml/min/1.73 m^2^), and insulin use (Figures 3–4 and Figures S1–S3). Study results suggested that previous hyperlipidemia and HbA1c level might alter the association between ln (10 × CMI) and MACEs. In particular, ln (10 × CMI) showed stronger predictive power for MACEs among T2DM patients with a history of hyperlipidemia or lower HbA1c levels. Additionally, eGFR level exerted a significant modifying effect on the relationship between ln (10 × CMI) and all-cause mortality.

### The additional predictive value of CMI for MACEs and all-cause mortality

C-statistics, NRI, and IDI were used to assess the predictive utility of the CMI for MACEs and all-cause mortality (Table 3). The integration of CMI into the standard risk model led to a significant enhancement in predictive performance for both MACEs and all-cause mortality among patients with T2DM. When CMI was added to the conventional model, the model's ability to reclassify risk and discriminate outcomes for MACEs was strengthened—yielding an NRI of 0.073 (95% CI: 0.045–0.102; *P* < 0.001) and an IDI of 0.004 (95% CI: 0.002–0.006; *P* < 0.001), the absolute C-index was elevated by 0.004. Considering the limited absolute increments of C-index, NRI and IDI for discrimination performance, supplementary calibration analysis was conducted to characterize the clinical predictive value conferred by incorporation of CMI. Calibration curves with 500-time bootstrap correction were plotted for pairwise comparison: the baseline traditional model exhibited slight departure of observed survival from ideal prediction at high predicted-risk segments. After adding CMI, the bias-corrected observed curves were substantially closer to the ideal reference line over the entire predicted probability range, with obvious correction of previous high-range calibration drift (Figure S4). Collectively, despite the modest numerical increments in discrimination metrics, the evident improvement of model calibration confirmed that CMI contributed to narrowing the gap between predicted MACE risk and real clinical event probability, providing robust evidence for its incremental clinical predictive utility in practical risk prediction. Comparable improvements were seen for all-cause mortality, CVD mortality, and nonfatal MI, though no meaningful gains were observed in the predictive capacity for nonfatal stroke.

**Table 3:** Additional predictive value of CMI for MACEs and all-cause mortality.

Model	C-index (95% CI)	*P*-value	NRI (95% CI)	*P*-value	IDI (95% CI)	*P*-value
MACEs						
Conventional model	0.662 (0.649, 0.675)		Ref		Ref	
Conventional model + ln (10 × CMI)	0.666 (0.653, 0.679)	0.0268	0.073 (0.045, 0.102)	< 0.0001	0.004 (0.002, 0.006)	< 0.0001
CVD mortality						
Conventional model	0.706 (0.685, 0.727)		Ref		Ref	
Conventional model + ln (10 × CMI)	0.711 (0.690, 0.732)	0.0743	0.106 (0.059, 0.135)	< 0.0001	0.002 (0.000, 0.005)	0.0070
Nonfatal MI						
Conventional model	0.657 (0.639, 0.675)		Ref		Ref	
Conventional model + ln (10 × CMI)	0.664 (0.646, 0.681)	0.0455	0.091 (0.049, 0.133)	< 0.0001	0.003 (0.001, 0.006)	< 0.0001
Nonfatal stroke						
Conventional model	0.647 (0.623, 0.672)		Ref		Ref	
Conventional model + ln (10 × CMI)	0.648 (0.624, 0.673)	0.6323	0.037 (-0.022, 0.085)	0.1400	0.000 (-0.000, 0.002)	0.2390
All-cause mortality						
Conventional model	0.673 (0.661, 0.686)		Ref		Ref	
Conventional model + ln (10 × CMI)	0.678 (0.664, 0.689)	0.0184	0.078 (0.046, 0.103)	< 0.0001	0.003 (0.001, 0.005)	< 0.0001

Conventional model: age, sex, CVD history, family history of heart disease, heart attack, or stroke, duration of diabetes, smoking, HbA1C, LDL-C, eGFR. CI, confidence interval; CMI, cardiometabolic index; CVD, cardiovascular disease; IDI, integrated discrimination improvement; MACEs, major adverse cardiovascular events; MI, myocardial infarction; NRI, net reclassification index.

### Sensitivity analysis

Sensitivity analyses were performed to evaluate the robustness of the primary findings. Given that lipid-lowering therapies may modify both CMI values and cardiovascular outcomes, we conducted a sensitivity analysis restricted to participants receiving statin therapy. The effect estimates obtained from this restricted cohort remained consistent with those from the primary analysis (Table S3).

### ROC-based cutoff point for ln (10 × CMI)

To improve the clinical practicability of CMI in individualized risk assessment and clinical decision-making, ROC curve analysis was performed to identify its optimal predictive cutoff for MACEs. The corresponding area under the ROC curve was calculated as 0.552. Using the maximum Youden index criterion, the optimal threshold of ln (10 × CMI) was determined to be 3.1598 (Figure S5).

## Discussion

CMI was initially utilized as a predictive marker for the risks of diabetes and CVD (17). However, research regarding whether CMI can predict MACEs in diabetic patients remains limited. The aim of this study is to explore the relationship among CMI, MACEs, and all-cause mortality in patients with T2DM. The findings indicate that the baseline CMI is a promising predictor of MACE and all-cause mortality in T2DM patients. After adjusting for confounding factors, elevated levels of CMI are independently associated with an increased risk of future MACEs and all-cause mortality in these patients. The integration of CMI into the conventional model, which encompasses traditional risk factors, improves the predictive capability for MACE and all-cause mortality in patients with T2DM. CMI can serve as a valuable indicator for predicting cardiovascular outcomes in patients with T2DM and enhancing risk stratification.

CMI is a non-invasive index integrating TG/HDL-C and WHtR, which reflects an individual's cardiovascular metabolic status (17, 28). WHtR serves as a valuable parameter for assessing central obesity and facilitates the identification of CVD (15). TG/HDL-C, in turn, enables the detection of metabolic disorders and CVD (14). A single risk factor may not fully reflect an individual's metabolic health status, whereas integrating multiple metabolic indicators enables a more accurate assessment of cardiovascular health risks. Existing evidence indicates that roughly 20% of cardiovascular events stem from mild metabolic derangements or central adiposity (29). CMI efficiently identifies such cryptic high-risk subjects to facilitate refined risk stratification and personalized intervention design. Previous studies have demonstrated associations between CMI and several conditions, including metabolic syndrome (28), non-alcoholic fatty liver disease (21), chronic obstructive pulmonary disease (23), heart failure (30), hypertension (19), depression (31), and IR (32). The findings demonstrate that the CMI serves as a robust predictor of metabolic diseases and patient prognosis. This study demonstrates that a higher CMI is significantly associated with an increased cumulative risk of MACEs and all-cause mortality in patients with T2DM. For each 1 SD increase in ln (10 × CMI), the risk of MACEs increases by 9%, and the risk of all-cause mortality increases by 10%.

In the fully adjusted model, while CMI exhibits a significant correlation with MACEs, no significant associations were observed between CMI and non-fatal stroke. Consistent with this finding, another study had reported no significant association between CMI and stroke (33). However, two population-based cross-sectional studies have documented a positive correlation between CMI and stroke risk (24, 34). One of these studies focused on adults aged ≥40 years, while the other targeted rural residents, limiting their generalizability to the broader population. Thus, the relationship between CMI and stroke warrants further investigation.

CMI integrates central obesity and dyslipidemia—two key factors contributing to elevated CVD risk (35). Central obesity is primarily characterized by the accumulation of visceral adipose tissue (VAT), which exhibits greater efficacy in predicting CVD risk compared to subcutaneous adipose tissue (SAT) (36). Increased VAT accumulation induces elevated production of pro-inflammatory cytokines, including interleukin-6 (IL-6) and tumor necrosis factor-α (TNF-α), which triggers systemic inflammation, impairs endothelial function, and promotes atherosclerosis development (37, 38). Visceral fat induces elevated circulating free fatty acid levels, which contribute to lipotoxicity and pancreatic β-cell dysfunction, thereby further exacerbating IR and dyslipidemia (39). In addition, an elevated TG/HDL-C ratio reflects marked lipoprotein metabolic dysfunction, which facilitates the generation of small, dense LDL particles. These particles frequently deposit within arterial walls, thereby contributing to atherosclerotic plaque formation (40). Lipid metabolism disorders may cause vascular damage through oxidative stress (41). Oxidative stress is caused by an imbalance between the production and clearance of reactive oxygen species, leading to cell and tissue damage (42). Lipid peroxidation is a major manifestation of oxidative stress. It not only directly damages vascular endothelial cells but also promotes inflammatory responses and the progression of atherosclerosis, leading to an increased risk of CVD (43). Central obesity and lipid metabolism disorders may act synergistically via shared metabolic pathways and pathological mechanisms to drive the onset and progression of CVD. Chronic inflammation induced by central obesity can exacerbate lipid metabolism disorders, while lipid metabolism disorders can further amplify IR and oxidative stress (44, 45). Therefore, as a comprehensive indicator, CMI can more holistically reflect an individual's metabolic health status. By integrating multiple metabolic abnormalities, CMI enhances the accuracy of cardiovascular disease risk prediction. The relationship between CMI and MACEs may be mediated through various complex mechanisms, including central obesity, lipid metabolism disorders, chronic inflammation, oxidative stress, and IR. These mechanisms not only independently promote cardiovascular disease progression but also interact synergistically to further amplify CVD risk.

It is well established that approximately 87.9% of myocardial infarctions are classified as type 1 myocardial infarction, which results from atherosclerotic plaque rupture, ulceration, or erosion with subsequent coronary thrombosis (46). In contrast, the etiological spectrum of ischemic stroke is notably different: only approximately 11 % of ischemic strokes are attributable to large-artery atherosclerosis, whereas cardioembolic and cryptogenic (undetermined) strokes are more common, accounting for approximately 34 % and 33 % of cases, respectively (47). Given that CMI primarily promotes atherosclerosis-driven plaque formation, its predictive performance for stroke risk may be inherently limited. Therefore, for stroke risk stratification, alternative biomarkers are needed. Previous studies have demonstrated significant associations between stroke risk and serum uric acid levels (48), as well as serum corin levels (49), underscoring the potential value of these biomarkers in stroke prediction.

In the subgroup analysis, CMI exhibited a stronger predictive capacity for MACEs and all-cause mortality among patients with lower HbA1c. This observation may be attributed to enhanced management of central obesity and dyslipidemia among patients with higher HbA1c levels. Furthermore, a more significant association was found between CMI and all-cause mortality among participants with lower eGFR values. Dyslipidemia is a common comorbidity during the progression of chronic kidney disease (CKD), occurring even in the early stages of eGFR decline. Elevated TG levels and reduced HDL-C levels have been identified as the primary components of dyslipidemia in CKD (50). Nevertheless, Multiple subgroup stratification and interaction testing increase the likelihood of chance-driven positive results. Since most subgroup analyses were *post-hoc* exploratory analyses rather than protocol-defined confirmatory tests, significant interactions observed for HbA1c and eGFR should be interpreted prudently and are insufficient to confirm genuine effect modification.

The strengths of this study include being the first to investigate the impact of CMI on future MACEs and all-cause mortality in patients with T2DM. Additional merits encompass a large cohort size and comprehensive follow-up of MACEs. Following thorough adjustment for cardiovascular risk factors, this study objectively validated the significant clinical value and potential utility of CMI as a biomarker for predicting MACEs and all-cause mortality in patients with T2DM. However, this study has several limitations. First, it constitutes a *post-hoc* analysis of the ACCORD and ACCORDION trials. Although potential confounding factors were adjusted for in the multivariate Cox regression analysis, residual confounding from unmeasured variables in the original trials may remain beyond our control. Second, the participants in the ACCORD and ACCORDION trials were T2DM patients at high CVD risk. Thus, additional research is required to verify the impact of CMI on MACEs in other populations. Finally, this study did not explore the impact of dynamic changes in CMI on MACEs and all-cause mortality, underscoring the need for further studies to evaluate its predictive value for cardiovascular adverse outcomes.

## Conclusion

In conclusion, this study confirms CMI can predict MACEs and all-cause mortality risks in T2DM patients. Further validation is needed before CMI, as a potential risk marker, is routinely applied to guide early intervention for adverse outcome reduction. Our results supply preliminary evidence for T2DM risk stratification, with their generalizability awaiting further verification.

## Data Availability

Publicly available datasets were analyzed in this study. This data can be found here: https://biolincc.nhlbi.nih.gov/studies/accord/.
