# Supplementary material for: Prognostic value of the cardiometabolic index for cardiovascular outcomes in patients with type 2 diabetes mellitus
Source: Front Nutr. 2026 Jul 24;13:1787383. doi: 10.3389/fnut.2026.1787383 (PMC13447206; doi:10.3389/fnut.2026.1787383)
Supplement: Supplementary file 1 [file Table_1.docx]

Table S1. Univariate analysis to predict MACEs

| Characteristics | HR | 95% CI | P-value |
| --- | --- | --- | --- |
| Ln (10 × CMI) | 1.23 | 1.16-1.30 | <0.0001 |
| Ln (10 × CMI) tertile |  |  |  |
| Low | 1.00 |  |  |
| Middle | 1.24 | 1.10-1.40 | 0.0003 |
| High | 1.47 | 1.31-1.65 | <0.0001 |
| Age | 1.04 | 1.03-1.04 | <0.0001 |
| Sex (Female) | 0.69 | 0.63-0.77 | <0.0001 |
| Race (Non-white) | 0.75 | 0.67-0.82 | <0.0001 |
| Treatment (Intensive glucose control) | 0.94 | 0.85-1.03 | 0.2584 |
| Education |  |  |  |
| Less than high school graduate | 1.00 |  |  |
| High school grad (or GED) | 0.83 | 0.72-0.96 | 0.0107 |
| Some college or technical school | 0.85 | 0.74-0.98 | 0.0252 |
| College graduate or more | 0.68 | 0.58-0.78 | <0.0001 |
| Living alone | 0.91 | 0.81-1.02 | 0.0894 |
| Depression | 1.25 | 1.13-1.39 | <0.0001 |
| CVD History | 2.40 | 2.18-2.63 | <0.0001 |
| Family history of heart disease, heart attack, or stroke | 1.22 | 1.11-1.34 | <0.0001 |
| Heart failure | 2.47 | 2.11-2.90 | <0.0001 |
| Previous hyperlipidemia | 1.09 | 0.99-1.21 | 0.0849 |
| Previous hypertension | 1.21 | 1.09-1.36 | 0.0007 |
| Smoking | 1.24 | 1.09-1.41 | 0.0011 |
| Duration of diabetes | 1.02 | 1.02-1.03 | <0.0001 |
| Proteinuria | 1.30 | 1.17-1.45 | <0.0001 |
| BMI | 1.00 | 0.99-1.01 | 0.4958 |
| SBP | 1.01 | 1.00-1.01 | <0.0001 |
| DBP | 0.99 | 0.98-0.99 | <0.0001 |
| Heart rate | 1.00 | 0.99-1.00 | 0.2475 |
| HbA1C | 1.12 | 1.08-1.17 | <0.0001 |
| Total plasma cholesterol | 1.00 | 1.00-1.00 | 0.0531 |
| TG | 1.00 | 1.00-1.00 | 0.0001 |
| VLDL | 1.00 | 1.00-1.00 | 0.0002 |
| LDL-C | 1.00 | 1.00-1.00 | 0.0334 |
| HDL-C | 0.98 | 0.98-0.99 | <0.0001 |
| ALT | 1.00 | 0.99-1.00 | 0.0061 |
| Serum creatinine | 2.68 | 2.25-3.19 | <0.0001 |
| eGFR | 0.99 | 0.99-1.00 | <0.0001 |
| Urinary albumin | 1.00 | 1.00-1.00 | <0.0001 |
| Urinary creatinine | 1.00 | 1.00-1.00 | 0.6737 |
| Medications |  |  |  |
| Diuretics | 1.33 | 1.21-1.46 | <0.0001 |
| ARB/ACEI | 1.01 | 0.92-1.12 | 0.7904 |
| CCB | 1.40 | 1.25-1.56 | <0.0001 |
| Beta-blockers | 1.69 | 1.54-1.86 | <0.0001 |
| Sulfonylureas | 0.92 | 0.84-1.01 | 0.0945 |
| Biguanides | 0.79 | 0.72-0.87 | <0.0001 |
| Meglitinides | 0.84 | 0.61-1.16 | 0.2898 |
| Thiazolidinediones | 0.82 | 0.73-0.93 | 0.0014 |
| Insulins | 1.45 | 1.32-1.59 | <0.0001 |
| Statins | 1.09 | 0.99-1.21 | 0.0722 |
| Fibrates | 1.12 | 0.93-1.35 | 0.2186 |
| Cholesterol absorption inhibitors | 1.18 | 0.86-1.63 | 0.3109 |

ALT, Alanine Aminotransferase; ARB, Angiotensin Receptor Blocker; ACEI, Angiotensin Converting Enzyme Inhibitors; BMI, Body Mass Index; CCB, Calcium Channel Blockers; CMI, Cardiometabolic Index； CVD, Cardiovascular Disease; DBP, Diastolic Blood Pressure; eGFR, estimated Glomerular Filtration Rate; GED, General education development; HDL-C, High-density Lipoprotein Cholesterol; LDL-C, Low-density Lipoprotein Cholesterol; MACEs, Major Adverse Cardiovascular Events; SBP, Systolic Blood Pressure; TC, Total Cholesterol; TG, Triglycerides; VLDL, Very Low-density Lipoprotein.

Table S2. Assessment of multicollinearity among adjustment variables

| **Variables** | **Tolerance** | **VIF** |
| --- | --- | --- |
| Ln (10 × CMI) | 0.786 | 1.271 |
| Age | 0.712 | 1.404 |
| Sex | 0.564 | 1.773 |
| Race | 0.803 | 1.246 |
| Education | 0.937 | 1.067 |
| Living alone | 0.958 | 1.044 |
| Depression | 0.931 | 1.074 |
| CVD History | 0.778 | 1.286 |
| Family history of heart disease, heart attack, or stroke | 0.944 | 1.060 |
| Heart failure | 0.930 | 1.076 |
| Previous hypertension | 0.913 | 1.095 |
| Smoking | 0.952 | 1.050 |
| Duration of diabetes | 0.743 | 1.346 |
| SBP | 0.601 | 1.665 |
| DBP | 0.558 | 1.791 |
| HbA1C | 0.924 | 1.082 |
| Total plasma cholesterol | 0.727 | 1.375 |
| ALT | 0.921 | 1.086 |
| Serum creatinine | 0.318 | 3.149 |
| eGFR | 0.382 | 2.620 |
| Urinary albumin | 0.930 | 1.075 |
| Diuretics | 0.872 | 1.147 |
| CCB | 0.940 | 1.063 |
| Beta-blockers | 0.805 | 1.242 |
| Sulfonylureas | 0.791 | 1.264 |
| Biguanides | 0.882 | 1.134 |
| Thiazolidinediones | 0.953 | 1.049 |
| Insulins | 0.635 | 1.575 |
| Statins | 0.819 | 1.222 |

ALT, Alanine Aminotransferase; CCB, Calcium Channel Blockers; CMI, Cardiometabolic Index； CVD, Cardiovascular Disease; DBP, Diastolic Blood Pressure; eGFR, estimated Glomerular Filtration Rate; SBP, Systolic Blood Pressure.

Table S3. Risk of MACEs and all-cause mortality based on ln (10 × CMI) in statin users

| Outcome | Events/n | Non-adjusted | | Model 1 | | Model 2 | | Model 3 | |
| --- | --- | --- | --- | --- | --- | --- | --- | --- | --- |
|  |  | HR (95% CI) | P-value | HR (95% CI) | P-value | HR (95% CI) | P-value | HR (95% CI) | P-value |
| **MACEs** |  |  |  |  |  |  |  |  |  |
| Ln (10 × CMI) |  | 1.27 (1.18, 1.37) | <0.0001 | 1.23 (1.13, 1.33) | <0.0001 | 1.18 (1.09, 1.29) | 0.0001 | 1.17 (1.07, 1.28) | 0.0004 |
| Low | 322/2212 | Ref |  | Ref |  | Ref |  | Ref |  |
| Middle | 416/2196 | 1.32 (1.14, 1.53) | 0.0002 | 1.24 (1.07, 1.44) | 0.0049 | 1.21 (1.04, 1.41) | 0.0126 | 1.21 (1.04, 1.41) | 0.0141 |
| High | 433/2009 | 1.56 (1.35, 1.81) | <0.0001 | 1.45 (1.24, 1.69) | <0.0001 | 1.38 (1.18, 1.62) | <0.0001 | 1.36 (1.15, 1.59) | 0.0002 |
| Per 1 SD |  | 1.21 (1.14, 1.28) | <0.0001 | 1.17 (1.10, 1.25) | <0.0001 | 1.14 (1.07, 1.22) | 0.0001 | 1.13 (1.06, 1.21) | 0.0004 |
| P for trend |  |  | <0.0001 |  | <0.0001 |  | <0.0001 |  | 0.0002 |
| **CVD mortality** |  |  |  |  |  |  |  |  |  |
| Ln (10 × CMI) |  | 1.31 (1.15, 1.48) | <0.0001 | 1.25 (1.09, 1.43) | 0.0016 | 1.22 (1.06, 1.41) | 0.0072 | 1.20 (1.04, 1.39) | 0.0158 |
| Low | 109/2212 | Ref |  | Ref |  | Ref |  | Ref |  |
| Middle | 145/2196 | 1.36 (1.06, 1.74) | 0.0156 | 1.28 (0.99, 1.65) | 0.0583 | 1.25 (0.97, 1.62) | 0.0841 | 1.25 (0.96, 1.61) | 0.0955 |
| High | 164/2009 | 1.73 (1.36, 2.20) | <0.0001 | 1.59 (1.23, 2.06) | 0.0004 | 1.55 (1.19, 2.03) | 0.0012 | 1.51 (1.15, 1.98) | 0.0028 |
| Per 1 SD |  | 1.23 (1.12, 1.35) | <0.0001 | 1.19 (1.07, 1.32) | 0.0016 | 1.17 (1.04, 1.31) | 0.0072 | 1.15 (1.03, 1.29) | 0.0158 |
| P for trend |  |  | <0.0001 |  | 0.0004 |  | 0.0012 |  | 0.0028 |
| **Nonfatal MI** |  |  |  |  |  |  |  |  |  |
| Ln (10 × CMI) |  | 1.35 (1.22, 1.49) | <0.0001 | 1.29 (1.15, 1.44) | <0.0001 | 1.25 (1.11, 1.41) | 0.0002 | 1.23 (1.09, 1.39) | 0.0006 |
| Low | 164/2212 | Ref |  | Ref |  | Ref |  | Ref |  |
| Middle | 223/2196 | 1.38 (1.13, 1.69) | 0.0016 | 1.28 (1.04, 1.58) | 0.0189 | 1.25 (1.02, 1.55) | 0.0344 | 1.24 (1.01, 1.54) | 0.0423 |
| High | 244/2009 | 1.70 (1.40, 2.08) | <0.0001 | 1.54 (1.25, 1.90) | <0.0001 | 1.48 (1.19, 1.84) | 0.0004 | 1.45 (1.16, 1.81) | 0.0010 |
| Per 1 SD |  | 1.26 (1.16, 1.36) | <0.0001 | 1.22 (1.12, 1.33) | <0.0001 | 1.19 (1.09, 1.30) | 0.0002 | 1.18 (1.07, 1.29) | 0.0006 |
| P for trend |  |  | <0.0001 |  | <0.0001 |  | 0.0004 |  | 0.0010 |
| **Nonfatal stroke** |  |  |  |  |  |  |  |  |  |
| Ln (10 × CMI) |  | 1.20 (1.03, 1.39) | 0.0163 | 1.16 (0.99, 1.36) | 0.0736 | 1.09 (0.92, 1.29) | 0.2987 | 1.11 (0.94, 1.31) | 0.2345 |
| Low | 86/2212 | Ref |  | Ref |  | Ref |  | Ref |  |
| Middle | 108/2196 | 1.27 (0.96, 1.69) | 0.0949 | 1.21 (0.90, 1.62) | 0.1996 | 1.21 (0.90, 1.62) | 0.2164 | 1.24 (0.92, 1.67) | 0.1634 |
| High | 113/2009 | 1.50 (1.14, 1.99) | 0.0044 | 1.42 (1.06, 1.92) | 0.0202 | 1.33 (0.98, 1.82) | 0.0705 | 1.37 (1.00, 1.88) | 0.0520 |
| Per 1 SD |  | 1.15 (1.03, 1.29) | 0.0163 | 1.12 (0.99, 1.27) | 0.0736 | 1.07 (0.94, 1.22) | 0.2987 | 1.08 (0.95, 1.24) | 0.2345 |
| P for trend |  |  | 0.0043 |  | 0.0199 |  | 0.0722 |  | 0.0539 |
| **All-cause mortality** |  |  |  |  |  |  |  |  |  |
| Ln (10 × CMI) |  | 1.18 (1.10, 1.27) | <0.0001 | 1.18 (1.09, 1.28) | <0.0001 | 1.14 (1.05, 1.24) | 0.0023 | 1.13 (1.03, 1.23) | 0.0080 |
| Low | 368/2212 | Ref |  | Ref |  | Ref |  | Ref |  |
| Middle | 412/2196 | 1.15 (0.99, 1.32) | 0.0590 | 1.12 (0.97, 1.29) | 0.1356 | 1.09 (0.94, 1.26) | 0.2314 | 1.09 (0.94, 1.26) | 0.2635 |
| High | 437/2009 | 1.37 (1.19, 1.57) | <0.0001 | 1.36 (1.17, 1.58) | <0.0001 | 1.30 (1.11, 1.51) | 0.0010 | 1.26 (1.08, 1.47) | 0.0037 |
| Per 1 SD |  | 1.14 (1.07, 1.20) | <0.0001 | 1.14 (1.07, 1.21) | <0.0001 | 1.11 (1.04, 1.18) | 0.0023 | 1.10 (1.02, 1.17) | 0.0080 |
| P for trend |  |  | <0.0001 |  | <0.0001 |  | 0.0009 |  | 0.0035 |

Model 1: adjusted for age, sex, race, education, living alone, depression, CVD history, family history of heart disease, heart attack, or stroke, heart failure, previous hypertension, duration of diabetes, smoking.

Model 2: adjusted for model 1 covariables plus SBP, DBP, HbA1C, TC, ALT, serum creatinine, eGFR, urinary albumin.

Model 3: adjusted for model 2 covariables plus the medications use, diuretics, CCB, beta-blockers, sulfonylurea, biguanides, thiazolidinediones and insulins.

Figure S1. Subgroup and interaction analyses of the association between ln (10 × CMI) and the risk of CVD mortality. Participants were stratified by sex, age (< 65 years and ≥ 65 years), race, CVD history, previous hyperlipidemia, previous hypertension, smoking status, duration of diabetes (< 10 years and ≥ 10 years), BMI (< 25 kg/m^2^ and ≥ 25 kg/m^2^), HbA1C (< 8.1% and ≥ 8.1%), eGFR (< 60 ml/min/1.73 m^2^, ≥ 60 ml/min/1.73 m^2^, < 90 ml/min/1.73 m^2^, and ≥ 90 ml/min/1.73 m^2^), and insulin use. BMI, Body Mass Index; CMI, cardiometabolic index; CVD, Cardiovascular Disease; eGFR, estimated Glomerular Filtration Rate.

Figure S2. Subgroup and interaction analyses of the association between ln (10 × CMI) and the risk of nonfatal MI. Participants were stratified by sex, age (< 65 years and ≥ 65 years), race, CVD history, previous hyperlipidemia, previous hypertension, smoking status, duration of diabetes (< 10 years and ≥ 10 years), BMI (< 25 kg/m^2^ and ≥ 25 kg/m^2^), HbA1C (< 8.1% and ≥ 8.1%), eGFR (< 60 ml/min/1.73 m^2^, ≥ 60 ml/min/1.73 m^2^, < 90 ml/min/1.73 m^2^, and ≥ 90 ml/min/1.73 m^2^), and insulin use. BMI, Body Mass Index; CMI, cardiometabolic index; CVD, Cardiovascular Disease; eGFR, estimated Glomerular Filtration Rate; MI, myocardial infarction.

Figure S3. Subgroup and interaction analyses of the association between ln (10 × CMI) and the risk of nonfatal stroke. Participants were stratified by sex, age (< 65 years and ≥ 65 years), race, CVD history, previous hyperlipidemia, previous hypertension, smoking status, duration of diabetes (< 10 years and ≥ 10 years), BMI (< 25 kg/m^2^ and ≥ 25 kg/m^2^), HbA1C (< 8.1% and ≥ 8.1%), eGFR (< 60 ml/min/1.73 m^2^, ≥ 60 ml/min/1.73 m^2^, < 90 ml/min/1.73 m^2^, and ≥ 90 ml/min/1.73 m^2^), and insulin use. BMI, Body Mass Index; CMI, cardiometabolic index; CVD, Cardiovascular Disease; eGFR, estimated Glomerular Filtration Rate.

Figure S4. Calibrated curves for MACE prediction. Black dashed line = ideal predicted survival; orange solid line = bias-corrected observed survival probability; blue vertical bars denote 95% CIs of grouped observed events. (A) Baseline conventional model in training cohort; (B) Baseline conventional model in validation cohort; (C) CMI-incorporated model in training cohort; (D) CMI-incorporated model in validation cohort.

Figure S5. ROC curve for ln (10 × CMI) in predicting MACE. ACU = 0.552, optimal cut-off = 3.1598.
